# Generation of Two Paclitaxel-Resistant High-Grade Serous Carcinoma Cell Lines With Increased Expression of P-Glycoprotein

**DOI:** 10.3389/fonc.2021.752127

**Published:** 2021-10-21

**Authors:** Mariana Nunes, Patrícia M. A. Silva, Ricardo Coelho, Carla Pinto, Albina Resende, Hassan Bousbaa, Gabriela M. Almeida, Sara Ricardo

**Affiliations:** ^1^Differentiation and Cancer Group, Institute for Research and Innovation in Health (i3S) of the University of Porto/Institute of Molecular Pathology and Immunology of the University of Porto (Ipatimup), Porto, Portugal; ^2^Institute of Biomedical Sciences Abel Salazar (ICBAS), University of Porto, Porto, Portugal; ^3^CESPU, Institute of Research and Advanced Training in Health Sciences and Technologies (IINFACTS), Gandra, Portugal; ^4^TOXRUN, Toxicology Research Unit, University Institute of Health Sciences, Polytechnic and University Cooperative (CESPU), Gandra, Portugal; ^5^Ovarian Cancer Research, Department of Biomedicine, University Hospital Basel and University of Basel, Basel, Switzerland; ^6^Interdisciplinary Centre of Marine and Environmental Research (CIIMAR), University of Porto, Porto, Portugal; ^7^Expression Regulation in Cancer Group, Institute for Research and Innovation in Health (i3S) of the University of Porto/Institute of Molecular Pathology and Immunology of the University of Porto (Ipatimup), Porto, Portugal; ^8^Faculty of Medicine from University of Porto (FMUP), Porto, Portugal

**Keywords:** high-grade serous carcinoma, ovarian cancer, chemoresistance, Paclitaxel, P-glycoprotein

## Abstract

Debulking surgery followed by chemotherapy are the standard of care for high-grade serous carcinoma. After an initial good response to treatment, the majority of patients relapse with a chemoresistant profile, leading to a poor overall survival. Chemotherapy regimens used in high-grade serous carcinomas are based in a combination of classical chemotherapeutic drugs, namely, Carboplatin and Paclitaxel. The mechanisms underlying drug resistance and new drug discovery are crucial to improve patients’ survival. To uncover the molecular mechanisms of chemoresistance and test drugs capable of overcoming this resistant profile, it is fundamental to use good cellular models capable of mimicking the chemoresistant disease. Herein, we established two high-grade serous carcinoma cell lines with intrinsic resistance to Carboplatin and induced Paclitaxel resistance (OVCAR8 PTX R C and OVCAR8 PTX R P) derived from the OVCAR8 cell line. These two chemoresistant cell line variants acquired an enhanced resistance to Paclitaxel-induced cell death by increasing the drug efflux capacity, and this resistance was stable in long-term culture and following freeze/thaw cycles. The mechanism underlying Paclitaxel resistance resides in a significant increase in P-glycoprotein expression and, when this drug efflux pump was blocked with Verapamil, cells re-acquired Paclitaxel sensitivity. We generated two high-grade serous carcinoma cell lines, with a double-chemoresistant (Carboplatin and Paclitaxel) phenotype that mimics the majority of tumor recurrences in ovarian cancer context. This robust tool is suitable for preliminary drug testing towards the development of therapeutic strategies to overcome chemoresistance.

## 1 Introduction

Late diagnosis and resistance to treatment are the main reasons for the high mortality rate of high-grade serous carcinoma (HGSC) patients (1, 2). There is still a lot to improve on these two issues to extend patients’ survival. On the treatment side, a successful debulking surgery (R0, defined as microscopic or no residual disease) is still the best prognostic factor for HGSC patients (3). After surgery, treatment with a combination of classical chemotherapy drugs, such as Carboplatin and Paclitaxel (PTX), is the standard of care (2, 4–6), although improvements in survival have been described in patients treated with Bevacizumab and PARP inhibitors (7–12).

Platinum-taxane-based chemotherapy is often used in cancer therapy in order to disrupt different cellular mechanisms involved in the regulation of the cell cycle to induce tumor cell death (13, 14). Unfortunately, in many HGSC patients, disease relapses within 6 months after first-line therapy and, in this setting, the median overall survival is approximately 12 months (15). The resistance mechanisms to platinum-based drugs are associated with a decrease in drug uptake (mediated by CTR1, CTR2, and OCT), an increase in drug efflux (mediated by ATP7A/ATP7B and MRP2), drug inactivation (mediated by GSH and Metallothionein), and alterations in DNA damage repair system (ERCC1, XPF, and BRCA1/BRCA2) (16). PTX resistance mechanisms are different and involve alterations in α- and β-tubulin, increased expression and activity of multidrug resistance (MDR) efflux transporters, overexpression of anti-apoptotic proteins, inhibition of apoptotic and tumor-suppressor proteins, and modulation of several cytokines, chemokines, and transcription factor pathways (17–25).

In the majority of established chemoresistant cell lines, Cisplatin is the drug mostly used for the induction of resistance (26). PTX-resistance induction is less commonly accessible, being established in gastric adenocarcinoma (OCUM-2M/PTX), breast cancer (MCF7/TAX), and prostate cancer (DU145-TxR, PC-3-TxR) models (27–29). In the ovarian cancer (OC) context, the majority of established chemoresistant cell lines are patient derived and have intrinsic resistance (30, 31). Also, A2780 and IGROV1 PTX-induced variants are available, but these cell lines correspond to endometrioid OC subtype (32–36).

Since HGSC is the most frequent epithelial OC histotype and double-resistant tumors are the major contributors to high mortality rate, we herein present a HGSC cell line model that combines intrinsic resistance to Carboplatin and acquired PTX chemoresistance. We generated two PTX-resistant cell lines derived from OVCAR8, with a stable acquired PTX resistance induced by P-glycoprotein (P-gp) overexpression. The development of these HGSC cell lines, capable of recapitulating the characteristics of double-resistant epithelial ovarian carcinoma, constitutes a key tool for the discovery of therapeutic alternatives to improve patients’ outcome.

## 2 Materials and Methods

### 2.1 Cell Line and Drugs

OVCAR8 was selected as a HGSC model, particularly since it is described as a Carboplatin-resistant OC cell line retrieved from a HGSC patient after a high-dose Carboplatin treatment (37). Parental OVCAR8 cell line was kindly provided by Doctor Francis Jacob, Gynecological Cancer Center and Ovarian Cancer Research, Department of Biomedicine, University Hospital Basel and University of Basel, Basel, Switzerland. Cells were grown in complete media, specifically, RPMI-1640 medium (ThermoFisher Scientific, Massachusetts, USA), supplemented with 10% (v/v) inactivated and filtered fetal bovine serum (FBS; Biowest, Nuaillé, France) and 1% (v/v) penicillin/streptomycin (ThermoFisher Scientific) and maintained at 37°C and 5% CO_2_. OVCAR8 was authenticated using short tandem repeat profiling and regularly tested for the absence of mycoplasma.

PTX was purchased from Selleckchem (Houston, Texas, USA), dissolved in dimethyl sulfoxide (DMSO; AppliChem, Barcelona, Spain) and stored at −80°C, according to the manufacturer’s instructions. Immediately prior to use, an aliquot was diluted at required concentrations.

### 2.2 Generation of OVCAR8 PTX R Cell Lines

**Figure 1:**
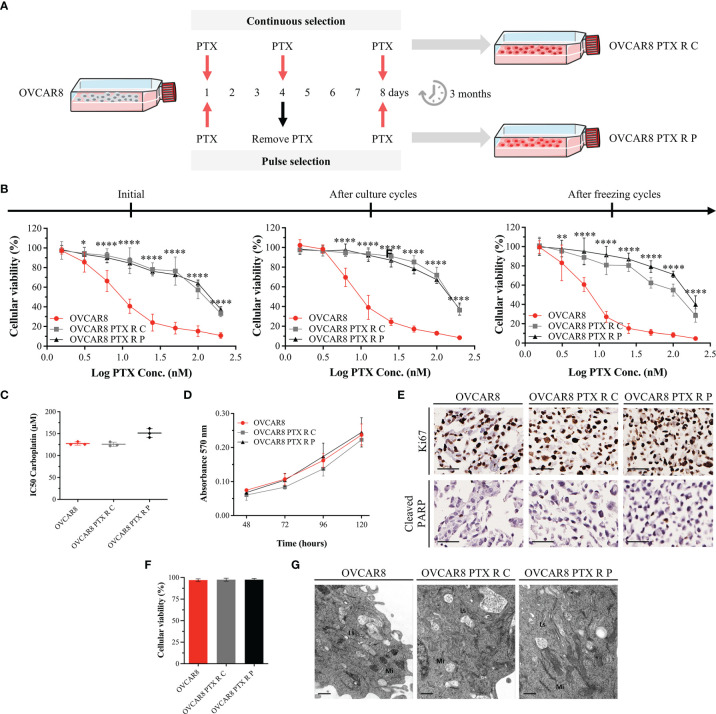
OVCAR8 PTX R Variants Demonstrate High Resistance to PTX. **(A)** Experimental design used to generate two OVCAR8 PTX R variants using two different treatment approaches: continuous (C) and pulse (P) selection. **(B)** Line chart showing cell viability for PTX in OVCAR8 and OVCAR8 PTX R cell lines obtained by PB assay after exposure to PTX (1.56 to 200 nM) for 48 h. **(C)** Dot plot showing IC50 for Carboplatin in OVCAR8 and OVCAR8 PTX R cell lines obtained by PB assay after exposure to Carboplatin (3.12 to 400 µM) for 48 h. **(D)** Line chart showing cell proliferation of OVCAR8 and OVCAR8 PTX R cell lines obtained by MTT assay for 120 h. **(E)** Representative immunocytochemistry images for Ki67 and cleaved-PARP of OVCAR8 and OVCAR8 PTX R cell lines after 96 h in culture. **(F)** Bar chart showing cell viability of OVCAR8 and OVCAR8 PTX R cell lines determined by trypan blue exclusion assay after 48 h in culture. **(G)** Ultrathin sections of OVCAR8 and OVCAR8 PTX R cell lines showing the ultrastructure of mitochondria (Mi), lysosomes (Ls), Golgi complex (asterisk), and rough endoplasmic reticulum (RER, arrows). All assays were done in triplicate in at least three independent experiments. Data are expressed as mean ± standard deviation and plotted using GraphPad Prism Software Inc. v6. Statistical analysis was performed using ordinary two-way ANOVA followed by Tukey’s multiple comparison test **(B, D)** and ordinary one-way ANOVA followed by Tukey’s multiple comparison test **(C**, **F)**, and values of *< 0.05; **< 0.001; ****< 0.0001 were considered statistically significant. Scale bar, 50 μm **(E)** and 0.5 μm **(G)** for immunocytochemistry images.

Two OVCAR8 PTX R variants were established in our laboratory from parental OVCAR8 by continuous (C) and pulse (P) exposure to a stepwise increasing PTX concentration (2 to 74.9 nM), for 3 months (details are shown in **Figure 1A**). The starting concentration used for PTX R induction corresponded to the IC10 value obtained from dose–response curves after exposing parental OVCAR8 to PTX (1.56 to 200 nM) for 48 h (**Supplementary Figure 1A**).

Initially, each OVCAR8 PTX R variant was maintained uninterruptedly in the presence of PTX for a continuous-selection strategy. After 1 month, two different approaches were adopted: (i) cells were exposed to PTX constantly (continuous-selection strategy) and (ii) cells were exposed to PTX, for 4 days followed by recovery in drug-free media for 4 days (pulse-selection strategy) for 2 months. Next, we tested if PTX R phenotype was stable in culture without drug. Hence, after 2 months of freezing cycles, cells were cultured in the absence of PTX medium supplementation (**Figure 1B**). Preston Blue (PB) assay was used to evaluate cell viability to PTX at different time points in order to confirm the maintenance of IC50 values after freezing cycles and persistent cultures.

### 2.3 Cell Viability Assay

To determine cell viability to PTX, a resazurin-based assay—Presto Blue (PB)—was performed, according to the manufacturer’s instructions. Briefly, 2 × 10^3^ cells/well were seeded into a 96-well plate in complete media and incubated at 37°C and 5% CO_2_. After 48 h, cells were treated with increasing concentrations of PTX (1.56 to 200 nM) and incubated under the same conditions. After 48 h, 50 μl of PrestoBlue™ Cell Viability Reagent 1× (ThermoFisher Scientific) was added and incubated for 45 min at 37°C and 5% CO_2_, protected from light. Fluorescence was measured (560 nm excitation/590 nm emission) using a Bio Tek SynergyTM 2 multi-mode microplate reader (BioTek, Vermont, USA). Treated cells were compared with control cells (considered 100% viable) containing 1% (v/v) of the vehicle (DMSO). The IC50 for parental OVCAR8 and PTX R variants was used to determine fold resistance, according to the following formula (38, 39):


$$\text{Fold Resistance}=\frac{\text{Resistant Cell Line IC}50}{\text{Parental Cell Line IC}50}$$

### 2.4 Proliferation Assay

To evaluate cell proliferation, MTT [3-(4.5-Dimethylthiazol-2-yl)-2,5-diphenyltetrazolium] assay was performed as previously described (40). Briefly, 1 × 10^3^ cells/well were seeded into a 96-well plate in complete media and incubated at 37°C and 5% CO_2_ up to 6 days. At each time point, 500 µg/ml of MTT dye reagent (Sigma-Aldrich, Missouri, USA) was added and incubated at 37°C and 5% CO_2_ for 3 h. Formazan crystals were dissolved in 100 µl of DMSO. Absorbance was measured (570 nm wavelength) using a Bio Tek SynergyTM 2 multi-mode microplate reader.

### 2.5 Viability Test

To differentiate viable and non-viable cells, trypan blue assay was performed. Cells at 80% confluence were diluted and mixed with a 0.4% trypan blue solution (ThermoFisher Scientific), placed in a Neubauer chamber, and counted in five counting grid squares, under a Leica DMi1 inverted phase contrast microscope (Leica Microsystems, Wetzlar, Germany), at 50× magnification.

### 2.6 Cell MicroArray Construction and Immunocytochemistry Expression Analysis

All conditions were arrayed in a Cell MicroArray (CMA) block designed and constructed as previously described (41, 42). Briefly, 2 × 10^5^ cells/well were seeded into six-well plates and incubated at 37°C and 5% CO_2_. After 48 h, cells were treated with 10 nM PTX and incubated under the same conditions. After 48 h, cells were scraped from culture dishes and fixed with 10% (v/v) neutral-buffered formalin (AppliChem). Cells were re-suspended in liquefied HistoGel™ (ThermoFisher Scientific), according to the manufacturer’s instructions, followed by standard histological processing and paraffin embedding. Finally, CMA block was constructed and sectioned with a microtome (43). To perform immunocytochemistry, slides were deparaffinized and hydrated. Next, heat-induced (98°C) antigen retrieval was performed with citrate buffer solution (1:100 at pH 6.0; ThermoFisher Scientific) or ethylenediamine tetraacetic acid (EDTA; 1:100; ThermoFisher Scientific) for 40 min. Endogenous peroxidase activity was blocked with hydrogen peroxide solution 3% (v/v) (ThermoFisher Scientific) for 10 min. Slides were incubated with a specific primary antibody (conditions described in **Supplementary Table 1**) and detected using a secondary antibody with horseradish peroxidase (HRP)-labeled polymer (Dako REAL™ EnVision™ Detection System Peroxidase/DAB+, Rabbit/Mouse) for 30 min. Visualization of the reaction was performed using diaminobenzidine according to the manufacturer’s instructions. Finally, nuclear staining with hematoxylin was performed and slides were dehydrated, clarified, and coverslipped using a permanent mounting medium for optical microscope analysis.

Immunocytochemistry results were evaluated by two independent observers (MN and SR) that register the staining pattern (nuclear, cytoplasm, or membrane) and the percentage of cells stained (0%, 1%–10%, 11%–25%, 26%–50%, 51%–75%, and 76%–100%).

### 2.7 Electronic Microscopy Analysis

To perform a qualitative assessment of cell ultrastructure, an electron microscopy analysis was performed. Briefly, for transmission electron microscopy and semithin section analysis, cultured cells were grown until 80% confluence, washed three times with phosphate buffer saline (PBS, ThermoFisher Scientific), gently scraped from culture dishes and fixed overnight in a glutaraldehyde (2.5%), paraformaldehyde (2%), and 0.1 M sodium cacodylate buffer (1:1) solution, at 4°C. After fixation, cells were pelleted by centrifugation and washed three times in 0.1 M sodium cacodylate buffer for 5 min. A post-fixation in 0.1 M sodium cacodylate buffered 2% osmium tetroxide was performed for 2 h at room temperature (RT). Then, cells were washed three times in distilled water for 5 min. The samples were incubated with 1% uranyl acetate for 30 min at RT, followed by three washes in distilled water for 5 min. Finally, cell pellet was imbedded in Histogel™, dehydrated in ethanol, and embedded in an epoxy resin. Ultrathin sections were stained and observed in a JEOL 100CXII transmission electron microscope (JEOL, Tokyo, Japan) operated at 80 kV, and images were digitally recorded using a CCD digital camera Orius 1100W (JEOL).

### 2.8 Apoptosis and Cell Cycle Analysis

To evaluate cell apoptosis and cell cycle distributions, Annexin V-FITC apoptosis detection kit™ (ThermoFisher Scientific) and propidium iodide (PI) staining were used, according to the manufacturer’s instructions. Briefly, 2 × 10^5^ cells/well were seeded into six-well plates and incubated at 37°C and 5% CO_2_. After 48 h, cells were treated with 10 nM PTX and incubated under the same conditions.

For apoptosis analysis, after 48 h, floating and adherent cells were collected using a cell dissociation buffer enzyme-free in PBS, pelleted by centrifugation (800 g, 5 min), filtrated through a 70-μm filter to obtain single-cell suspensions, and suspended in 195 µl of binding buffer. Next, 5 µl of Annexin V-FITC was added and incubated for 10 min, protected from light. Cells were washed, resuspended in 190 µl of binding buffer, and 10 µl of PI (20 μg/ml) was added and cells were incubated for 1 min, protected from light.

For cell cycle, following 24 h, floating and adherent cells were collected, pelleted by centrifugation, and resuspended in PBS. Cells were fixed with 70% (v/v) cold ethanol and incubated at 4°C for 30 min. Next, cells were pelleted by centrifugation, resuspended in PBS containing PI (250 µg/ml), and RNase A (100 µg/ml) and incubated at 4°C for 15 min, protected from light.

Fluorescence from both assays was assessed by BD FACS Canto™ II flow cytometer and data were analyzed by FlowJo software v10.0.7 (Ashland, Oregon, USA).

### 2.9 Time-Lapse Microscopy, Image Acquisition, and Processing

Live-cell imaging experiments were performed as previously described (44). Briefly, 1.2 × 10^5^ cells were seeded into LabTek II chambered cover glass (Nunc, New York, USA) containing 1 ml of culture medium and incubated at 37°C with 5% CO_2_. After 24 h, cells were treated with 20 nM PTX in RPMI without phenol red and supplemented with 5% FBS. Images were captured at 5-min intervals up to 72 h under differential interference contrast (DIC) optics, with a 63× objective under an Axio Observer Z.1 SD inverted microscope, equipped with an incubation chamber at 37°C and 5% CO_2_. Movies were generated from time-lapse images using ImageJ 1.4v software (Rasband, W.S., ImageJ, U.S. National Institutes of Health, Bethesda, Maryland, USA). The mitosis duration and the number of cells arrested at mitosis, dead by apoptosis, or bypassing cytokinesis were scored.

### 2.10 Anoikis Resistance Assay

To evaluate anoikis resistance, an aggregate formation assay was performed as previously described (40). Briefly, 1 × 10^6^ cells/well were seeded into polyHEMA (Poly2-hydroxyethyl methacrylate; Sigma-Aldrich)-coated plates and incubated at 37°C and 5% CO_2_ up to 15 days. At each time point (5, 10, and 15 days), floating cells were collected and dissociated using trypsin, pelleted by centrifugation, and resuspended in PBS. Cell suspensions were incubated with DAPI (1 µg/ml) for 2 min. Fluorescence was assessed by BD FACS Canto™ II flow cytometer, and data were analyzed by FlowJo software v10.0.7.

### 2.11 Colony Formation Assay

To evaluate clonogenic capacity, 1 × 10^3^ cells/well were seeded into six-well plates and incubated at 37°C and 5% CO_2_. Following 24 h, cells were treated with 10 nM PTX and incubated under the same conditions. After 48 h, media was replaced, and cells were allowed to recover for 8 days. Surviving colonies were fixed and stained as previously described (45). The percentage of cell survival was determined according to the following formula:


$$\text{Cell Survival }(\%)=\frac{\text{Colonies Number in Control}\times 100\%}{\text{Colonies Number in Treatment}}$$

### 2.12 Wound Healing Assay

To analyze migration capacity, cell monolayers at confluence cultured in six-well plates were serum starved overnight and incubated at 37°C and 5% CO_2_. Then, monolayers were washed twice with PBS and scratched in half with a sterile 200-µl pipette tip. To monitor cell migration, a phase contrast microscopy image was taken at 0, 24, and 48 h. The quantification of wound closure was performed by measuring the unmigrated remaining area using ImageJ 1.4v software.

### 2.13 RNA Isolation and Quantitative Real-Time PCR

Total RNA was extracted from cells with PureZOL™ RNA Isolation Reagent (Bio-Rad Laboratories, California, USA), according to the manufacturer’s instructions. cDNA synthesis was performed with iScript™ cDNA Synthesis Kit (Bio-Rad), using total cell RNA as template, following supplier’s instructions. For real-time PCR, cDNA was amplified using iQ™ SYBR Green Supermix Kit (Bio-Rad). Primers for P-gp were as follows: forward: 5’-GCCAAAGCCAAAATATCAGC-3’ and reverse: 5’-TTCCAATGTGTTCGGCATTA-3’; GAPDH: forward: 5’-ACAGTCCAGCCGCATCTTC-3’ and reverse: 5’-GCCCAATACGACCAAATCC-3’; and β-Actin, used as housekeeping gene: forward: 5’-AAT CTG GCA CCA CAC CTT CTA-3’ and reverse 5’-ATA GCA CAG CCT GGA TAG CAA-3’. Data were acquired with CFX Manager™ Software v1.0 (Bio-Rad) and results were analyzed according to ΔCT.

### 2.14 Cell Extracts and Western Blotting

Total cell protein extracts and Western blotting were performed as previously described (46). Membrane was incubated with primary (**Supplementary Table 1**) and secondary antibodies [anti-rabbit horseradish peroxidase (Vector Laboratories, Burlingame, USA) and anti-mouse horseradish peroxidase (Sigma-Aldrich), diluted at 1:1,000 or 1:4,000, respectively]. The protein signal intensity was quantified using ImageJ 1.4v software and normalized against α-tubulin expression levels.

### 2.15 Rhodamine 123 Accumulation Assay

To evaluate P-gp functional activity, a total of 1 × 10^5^ cells of OVCAR8 and OVCAR8 PTX R were seeded into six-well plates and incubated at 37°C and 5% CO_2_. After 24 h, 1 µM of the fluorescent P-gp substrate Rhodamine 123 (RH-123; Sigma-Aldrich) was added, in the presence or absence of 20 µM Verapamil (Sigma-Aldrich)—P-gp inhibitor—and incubated under the same conditions. After 1 h, cells were harvested and washed twice with ice-cold PBS, and cell pellet was gently re-suspended in ice-cold PBS for analysis. The mean fluorescence intensity (MFI) was assessed by BD Accuri C6™ II flow cytometer (BD Biosciences, California, USA), and data were analyzed by BD Accuri C6 Plus Software, version 1.0.27.1 (BD Biosciences).

### 2.16 SRB Assay

To determine cell viability to PTX after Verapamil incubation, a Sulforhodamine B (SRB) assay was performed. Briefly, 5 × 10^6^ cells/well were seeded in 96-well plates and incubated at 37°C and 5% CO_2_. After 24 h, cells were treated with 10-fold serial dilutions of PTX, in the presence or absence of 10 µM Verapamil and incubated under the same conditions. In parallel, cells were treated with equivalent amount of vehicle (DMSO) up to 0.25% concentration. Then, cells were fixed with 50% (m/v) trichloroacetic acid (Merck Millipore, Darmstadt, Germany) for 1 h, washed with distilled water, and stained with 0.1% (m/v) SRB in acetic acid (Sigma-Aldrich) for 30 min at RT. After washing with 1% (v/v) acetic acid aquae solution (Merck Millipore), plates were left to dry at RT followed by SRB complex solubilization with 10 mM Tris-Base buffer (Sigma-Aldrich) for 30 min. Absorbance was measured (515 nm wavelength) using a Bio Tek SynergyTM 2 multi-mode microplate reader. The IC50 of PTX, in the presence or absence of Verapamil, was determined as described above in *Section 2.3*.

### 2.17 Statistical Analysis

All assays were done in triplicate with at least three independent experiments. Data were expressed as mean ± standard deviation (SD), statistical analysis was carried out in GraphPad Prism Software Inc. v6 using ordinary one-way or two-way ANOVA followed by Tukey’s or Šídák’s multiple comparison test, and values of *<0.05; **<0.001; ***<0.0005; ****<0.0001 were considered statistically significant.

## 3 Results

### 3.1 OVCAR8 PTX R Variants Demonstrate High Resistance to PTX

OVCAR8 and OVCAR8 PTX R cell lines were treated with increasing concentrations of PTX, and cell viability was evaluated by PB assay. Cell viability of OVCAR8 cell line exposure to 10 nM PTX was 51.52% ± 7.69%, whereas for OVCAR8 PTX R C and OVCAR8 PTX R P variants, it was 89.16% ± 6.62% and 86.79% ± 4.25%, respectively (**Figure 1B**). A significant increase in IC50 PTX was observed for OVCAR8 PTX R C (128.97 ± 6.48 nM, *p* < 0.001) and OVCAR8 PTX R P (152.80 ± 6.51 nM, *p* < 0.001) variants when compared to OVCAR8 cell line (10.51 ± 1.99 nM), demonstrating a 12.27-fold and 14.54-fold increase in resistance, respectively (**Supplementary Figure 1B**).

In order to assess the stability of acquired resistance in both OVCAR8 PTX R variants, sensitivity to PTX was assessed after two continuous months in culture and after cryopreservation cycles, without PTX medium supplementation. Our results show that OVCAR8 PTX R variants have a long-term stability without drug supplementation and retain their resistant phenotype following freeze/thawing cycles (**Figure 1B** and **Supplementary Figure 1B**).

Additionally, we evaluated if OVCAR8 PTX R variants increased resistance to Carboplatin, since parental OVCAR8 cell line was described as a Carboplatin chemoresistant model. Our results show no significant differences in IC50 for Carboplatin concentrations in OVCAR8 PTX R C (125.74 ± 3.60 nM) and OVCAR8 PTX R P (151.26 ± 10.17 nM, *p* < 0.05) when compared to OVCAR8 cell line (127.49 ± 4.76 nM) (**Figure 1C**).

To further evaluate the PTX R phenotype, we performed cell proliferation, apoptosis, and viability assays. By MTT assay, no significant increase in proliferation was observed in OVCAR8 PTX R variants compared to OVCAR8 cell line, at 120 h in culture (**Figure 1D**). Accordingly, by immunocytochemistry, no significant differences were observed in proliferation (75%–100% of Ki67 staining) and apoptosis (1%–10% of cleaved-PARP staining) for OVCAR8 PTX R variants when compared to OVCAR8 cell line after 48 h in culture (**Figure 1E**). Trypan blue exclusion assay for assessing viability indicated no significant differences between OVCAR8 PTX R variants (both 97.50% viable) when compared to OVCAR8 cell line (97.50% viable) (**Figure 1F**).

To assess ultrastructural alterations in OVCAR8 PTX R variants, we performed qualitative analysis of several transmission electron microscopy micrographs. No significant ultrastructural differences were observed between OVCAR8 and OVCAR8 PTX R cell lines, which revealed similar ultrastructure aspects such as numerous dense mitochondria, developed Golgi apparatus and rough endoplasmic reticulum, and multiple lysosomes (**Figure 1G**).

### 3.2 OVCAR8 PTX R Variants Overcome PTX-Induced G2/M Arrest and Apoptosis

**Figure 2:**
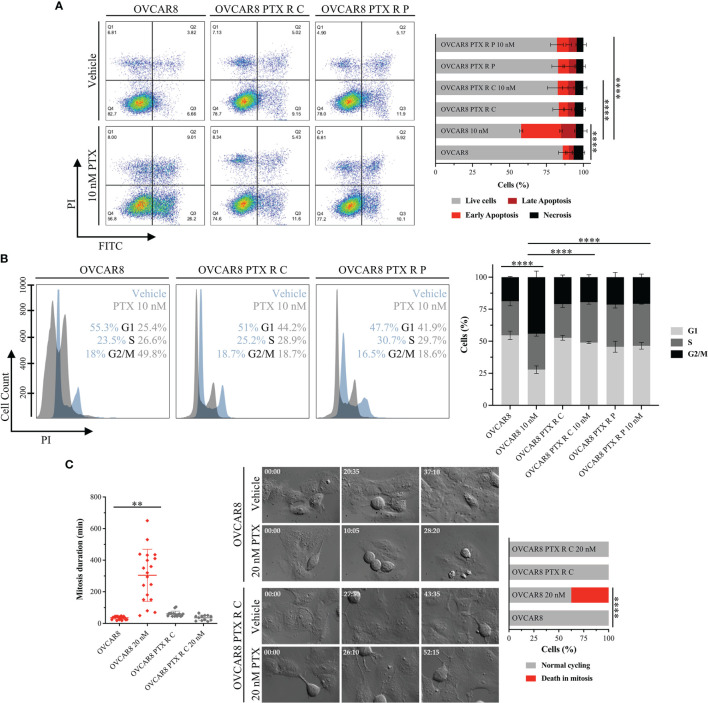
OVCAR8 PTX R Variants Overcome PTX-Induced G2/M Arrest and Apoptosis. **(A)** Representative flow cytometry dot plots and bar chart showing the percentage of cell populations—necrosis (Q1), late apoptosis (Q2), early apoptosis (Q3), and live cells (Q4)—of OVCAR8 and OVCAR8 PTX R cell lines using Annexin V/PI staining, after exposure to 10 nM PTX for 48 h. **(B)** Representative flow cytometry histogram and bar chart for cell cycle distribution (G1, S, and G2/M) of OVCAR8 and OVCAR8 PTX R cell lines using propidium iodide (PI) staining, after exposure to 10 nM PTX for 24 h. **(C)** Representative time-lapse images and corresponding dot plot for mitosis duration and bar chart for quantification of cell fate (normal cycling or death in mitosis) in OVCAR8 and OVCAR8 PTX R cell lines, after exposure to 20 nM PTX for 72 h. All assays were done in triplicate in at least three independent experiments. Flow cytometry was performed using BD FACS Canto™ II (BD Biosciences) flow cytometer and data were analyzed by FlowJo software v10.0.7. Data are expressed as mean ± standard deviation and plotted using GraphPad Prism Software Inc. v6. Statistical analysis was performed using ordinary one-way ANOVA followed by Tukey’s multiple comparison test **(A–C)** and two-way ANOVA followed by Tukey’s multiple comparison test **(C)**, and values of **< 0.001; ****< 0.0001 were considered statistically significant. Scale bar, 100 μm for microscopic images.

Levels of PTX-induced apoptosis were determined by the Annexin V-FITC apoptosis detection kit™ using flow cytometry. Upon 48 h incubation with 10 nM PTX, a significant (*p* < 0.0001) increase in PTX-induced apoptosis was observed on OVCAR8 cell line (37.01% ± 1.63%) when compared with the nontreated control (7.40 ± 2.48%) (**Figure 2A**). In contrast, after 10 nM PTX exposure, our results show a significant decrease (*p* < 0.0001) in PTX-induced apoptosis in OVCAR8 PTX R C and P variants (11.74% ± 4.58% and 13.38% ± 2.33%, respectively) when compared to OVCAR8 cell line (37.01% ± 1.63%) (**Figure 2A**).

Cell cycle distribution was assessed through PI staining by flow cytometry. After 10 nM PTX exposure, a significant decrease (*p* < 0.0001) in the percentage of cells in G1 phase (54.61 ± 3.32% *vs.* 27.84 ± 2.89%) and accumulation of cells in G2/M phase (18.77 ± 0.56% *vs.* 44.18 ± 4.75%) was observed in the OVCAR8 cell line when compared to control (**Figure 2B**). On the other hand, after 10 nM PTX exposure, OVCAR8 PTX R variants showed no significant changes in G1, S, and G2/M subpopulations when compared to control (**Figure 2B**). After 10 nM PTX exposure, a significant difference (*p* < 0.0001) in the percentage of cells in G1 phase was found in OVCAR8 PTX R C and P variants (48.92% ± 0.84% and 46.41% ± 2.62%, respectively) when compared to parental cell line (27.84% ± 2.89%) (**Figure 2B**). Likewise, a significant difference (*p* < 0.0001) in the G2/M population was found in OVCAR8 PTX R C and P variants (19.57% ± 1.94% and 20.82% ± 2.45%, respectively) when compared to the parental cell line (44.18% ± 4.75%) (**Figure 2B**). A significant increase (*p* < 0.001) was also observed in sub-G1 fraction (debris and apoptotic cells) for the OVCAR8 cell line (3.11% ± 0.91% *vs.* 7.60% ± 1.16%) and the OVCAR8 PTX R P variant (2.41% ± 0.79% and 7.15% ± 0.76%) when compared to controls (**Supplementary Figure 2A**) after exposure to 10 nM of PTX. OVCAR8 and OVCAR8 PTX R cell lines presented a similar proportion of polyploid (>4C) population (**Supplementary Figures 2A, B**).

To further characterize the behavior of OVCAR8 and OVCAR8 PTX R C cell lines, we analyzed mitosis duration and cell fate by live-cell imaging using time-lapse DIC microscopy. We found that the OVCAR8 PTX R C variant spent 55.33 ± 7.66 min in mitosis, from nuclear envelope breakdown to anaphase onset, similarly to what was observed in the OVCAR8 cell line that spent 39.35 ± 16.70 min (**Figure 2C**). Under 20 nM PTX exposure, the mitosis duration in OVCAR8 PTX R C variant is similar to that in untreated cells (33.00 ± 15.18 min) (**Figure 2C**) contrasting with the significant increase (*p* < 0.001) exhibited by the OVCAR8 cell line (331.29 ± 106.82 min). After 20 nM PTX exposure, the survival fate (outcome of cells delayed in mitosis under PTX exposure) analysis showed that 37.5% of parental OVCAR8 cells died in mitosis after mitotic arrest, and 62.5% of cells underwent normal cycling (**Figure 2C**). In contrast, OVCAR8 PTX R C variant undertook normal and multiple mitosis (**Figure 2C**).

### 3.3 OVCAR8 PTX R Variants Retain Parental OVCAR8 Features: High Anoikis Resistance, Colony Formation, and Migration Capacity

**Figure 3:**
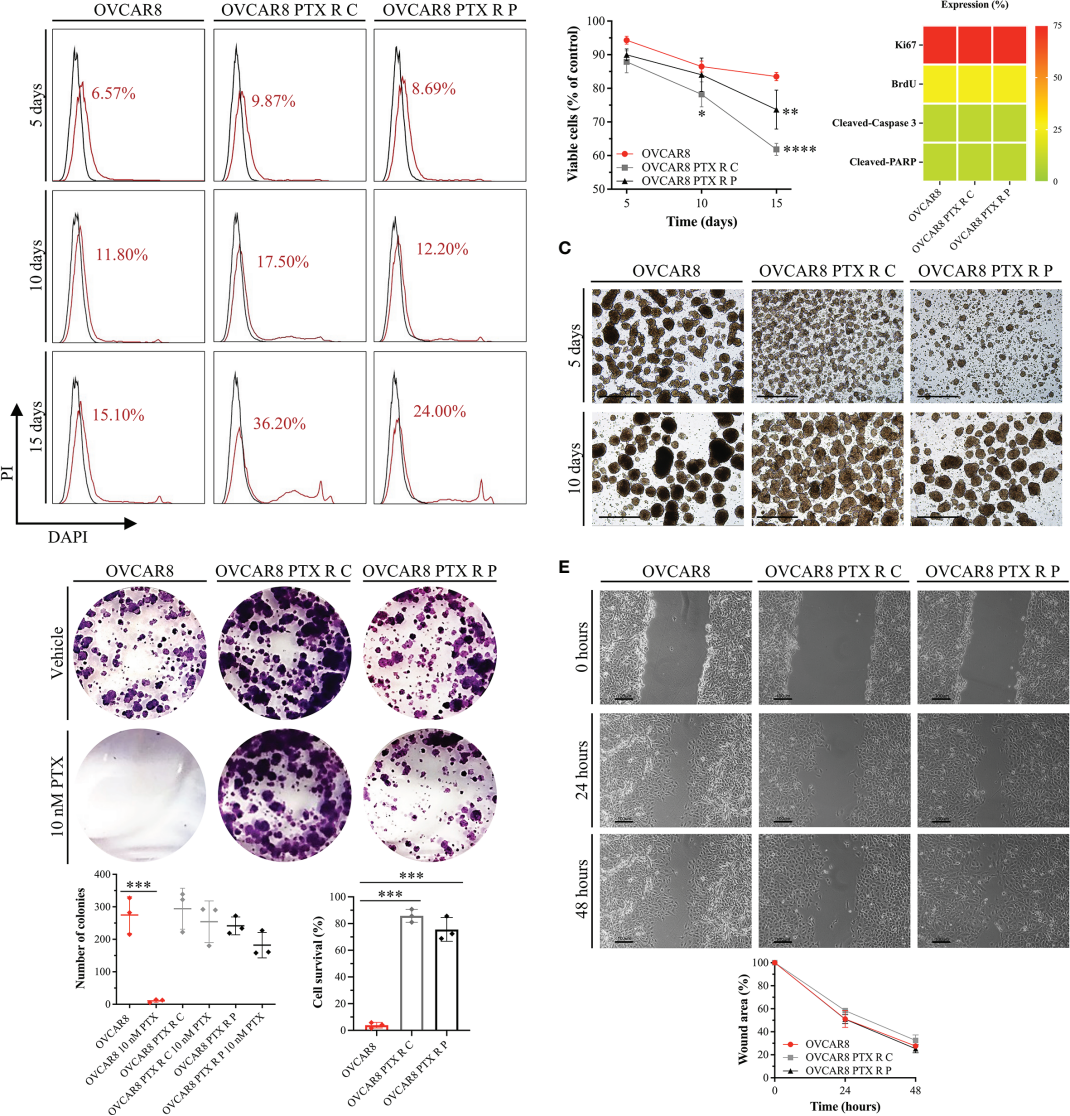
OVCAR8 PTX R Variants Reveal a High Anoikis Resistance, Colony Formation, and Migration Capacity. **(A)** Representative flow cytometry histograms for cell-detachment-induced apoptosis (anoikis) and line chart showing percentage of viable cells (DAPI negative cells) for OVCAR8 and OVCAR8 PTX R cell lines, for 5, 10, and 15 days. Unstained (black) and percentage of DAPI-stained cells (dead cells, red). DAPI positivity percentage is mentioned in each histogram. **(B)** Proliferation (Ki67 and BrdU) and apoptosis (cleaved-PARP and cleaved-caspase 3) expression for OVCAR8 and OVCAR8 PTX R cell lines were evaluated by immunocytochemistry and cultured after 15 days in low adhesion culture conditions. **(C)** Representative images of OVCAR8 and OVCAR8 PTX R cell lines, cultured after 5 and 10 days in low adhesion culture conditions. **(D)** Representative wells from colony-forming assay and dot plot showing quantification of number of cell colonies (*n* = 9 wells) and percentage of cell survival for OVCAR8 and OVCAR8 PTX R cell lines after 8 days in culture. **(E)** Representative phase-contrast microscopy images for wound healing assay and line chart showing wound area quantification for OVCAR8 and OVCAR8 PTX R cell lines at 0, 24, and 48 h. All assays were done in triplicate in at least three independent experiments. Data are expressed as mean ± standard deviation and plotted using GraphPad Prism Software Inc. v6. Statistical analysis was performed using two-way ANOVA followed by Tukey’s multiple comparison test **(A, E)** and ordinary one-way ANOVA followed by Tukey’s multiple comparison test **(D)**, and values of *< 0.05; **< 0.001; ***< 0.0005; ****< 0.0001 were considered statistically significant. Scale bar, 20 μm **(C)** and 100 μm **(E)** for microscopic images.

To assess anoikis (cell detachment-induced apoptosis) resistance, an aggregate formation assay was performed and evaluated by flow cytometry. Our results for anoikis resistance demonstrate no significant differences in cell viability (DAPI negative cells) for OVCAR8 PTX R C (87.88% ± 3.29% and 78.20% ± 3.73%) and OVCAR8 PTX R P variants (89.96% ± 1.74% and 84.03% ± 4.97%) when compared to parental OVCAR8 cell line (94.32% ± 1.15% and 86.47% ± 1.66%), upon 5 and 10 days of culture, respectively (**Figure 3A**). Moreover, no significant differences were observed in the percentage of positive cells for Ki67 (76%–99%), BrdU (11%–25%), cleaved caspase 3 (1%–10%), and cleaved PARP (1%–10%) for OVCAR8 PTX R variants when compared to the parental OVCAR8 cell line (**Figure 3B**). Additionally, we demonstrate that aggregation capacity was partially affected, since different and irregular aggregates were formed in OVCAR8 PTX R cells when compared to OVCAR8 (**Figure 3C**).

To evaluate clonogenic capacity, we performed a colony-forming assay. Our results show that after 10 nM PTX exposure, a significant decrease (*p* < 0.0005) occurs in the number of colonies in the OVCAR8 cell line (274.78 ± 49.27 *vs.* 10.56 ± 8.59) when compared to the untreated control (**Figure 3D**). One the other hand, after 10 nM PTX exposure, no significant differences in colony formation capacity were observed in OVCAR8 PTX R C (294.22 ± 57.45 *vs.* 254.00 ± 58.68) and OVCAR8 PTX R P variants (241.44 ± 24.44 *vs.* 182.11 ± 44.29) when compared to untreated control (**Figure 3D**). In fact, the percentage of cell survival was significantly higher (*p* < 0.0005) in OVCAR8 PTX R C (85.80% ± 4.80%) and OVCAR8 PTX R P variants (74.92% ± 7.72%) when compared to the parental OVCAR8 cell line (3.98% ± 2.08%) (**Figure 3D**).

To evaluate migration capacity, we performed a wound healing assay. Our results display no significant differences on migration behavior for OVCAR8 PTX R C (32.48% ± 4.79%) and OVCAR8 PTX R P variants (25.27% ± 3.55%) when compared to the OVCAR8 cell line (27.63% ± 0.81%) at 48 h (**Figure 3E**).

### 3.4 OVCAR8 PTX R Variants Acquire a New Phenotype With P-gp Overexpression

**Figure 4:**
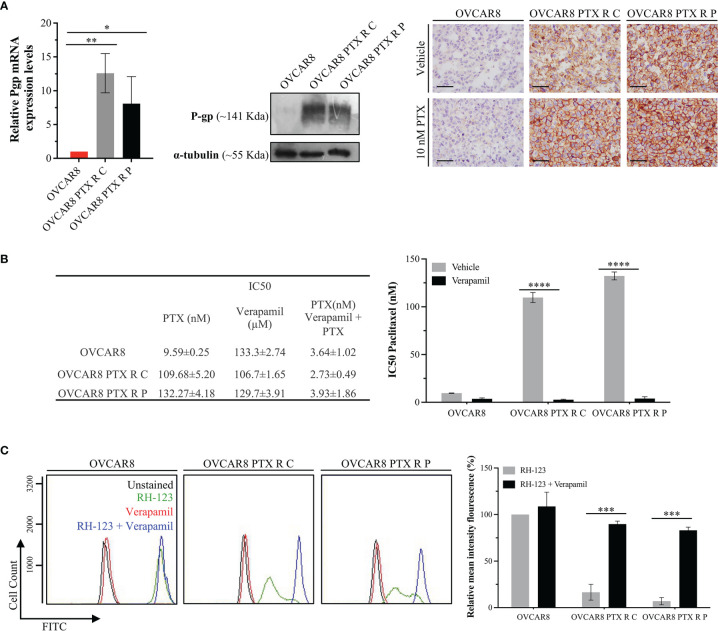
OVCAR8 PTX R Variants P-gp Overexpression. **(A)** Bar chart showing relative P-gp mRNA expression levels in OVCAR8 and OVCAR8 PTX R cell lines determined by qRT-PCR with β-Actin used as housekeeping gene. Representative Western blot showing P-gp expression in OVCAR8 and OVCAR8 PTX R cell lines using α-tubulin as loading control. Representative immunocytochemistry images for P-gp expression in OVCAR8 and OVCAR8 PTX R cell lines, after exposure to 10 nM PTX for 48 h. **(B)** Table showing IC50 PTX (in the presence and absence of 10 µM Verapamil) and IC50 Verapamil for OVCAR8 and OVCAR8 PTX R cell lines obtained by SRB assay for 48 h. Bar chart showing IC50 PTX for OVCAR8 and OVCAR8 PTX R cell lines, obtained by SRB assay after exposure to 10-fold serial dilutions of PTX, in the presence or absence of 10 µM of Verapamil for 48 h. **(C)** Representative flow cytometry histogram and respective bar chart showing RH-123 accumulation using FITC-A intensity in untreated (unstained, black) and RH-123-treated (stained, green) cells in the presence (blue) or absence (red) of P-gp inhibitor (Verapamil) for OVCAR8 and OVCAR8 PTX R cell lines. All assays were done in triplicate in at least three independent experiments. Data are expressed as mean ± standard deviation and plotted using GraphPad Prism Software Inc. v6. Statistical analysis was performed using ordinary two-way ANOVA followed by Tukey’s multiple comparison test **(A)** or Šídák’s multiple comparison test **(B, C)**, and values of *< 0.05; **< 0.001; ***< 0.0005; ****< 0.0001 were considered statistically significant. Scale bar, 500 μm for immunocytochemistry images.

P-gp encoded by *MDR1* is abundantly expressed in cellular membrane, where it functions as a multidrug efflux pump that transports a wide range of structurally different substrates, including PTX (47). P-gp overexpression is the main mechanism of resistance to chemotherapeutics, being responsible for pumping the drug out of the cells, resulting in low intracellular concentration of the drugs and survival of cancer cells (48, 49). P-gp expression was evaluated at mRNA and protein levels, by qRT-PCR, Western blot, and immunocytochemistry. From qRT-PCR analysis, we found an increase in P-gp expression on OVCAR8 PTX R C (12.60 ± 2.90-fold increase) and OVCAR8 PTX R P variants (8.08 ± 4.00-fold increase) when compared to OVCAR8 cell lines (**Figure 4A**). This result was confirmed at the protein level by Western blot and immunocytochemistry that revealed a high percentage of cells positive for P-gp in OVCAR8 PTX R variants (76%–100% positive cells) when compared to the OVCAR8 cell line (negative/residual expression) (**Figure 4A**).

To confirm if P-gp upregulation is the chemoresistance mechanism in OVCAR8 PTX R variants, we determined the IC50 PTX in the presence and absence of Verapamil, a well-known P-gp inhibitor. We found that the IC50 of PTX decreased in OVCAR8 PTX R C (40-fold) and OVCAR8 PTX R P variants (33-fold) when Verapamil was added (**Figure 4B**). We also analyzed by flow cytometry the activity of P-gp through the evaluation of the mean fluorescence intensity (MIF) of cells exposed to RH-123, a known P-gp fluorescent substrate. The percentage of MIF is proportional to intracellular uptake and extracellular efflux of RH-123. We found that intracellular accumulation of RH-123 was very low for OVCAR8 PTX R C and OVCAR8 PTX R P variants (16.57% ± 8.46% and 6.99% ± 3.91%, respectively) when compared to the parental OVCAR8 cell line (considered 100%). Moreover, in the presence of Verapamil, the relative MIF significantly increased (*p* < 0.0005) for OVCAR8 PTX R C (89.73% ± 3.22%) and OVCAR8 PTX R P variants (83.14% ± 3.30%) when compared to control, demonstrating an accumulation of RH-123 due to P-gp pump inhibition (**Figure 4C**).

## 4 Discussion

Surgery and chemotherapy remain the two major pillars in oncology treatments. Unfortunately, tumor recurrences and multidrug-resistant phenotype are common phenomena in HGSC context. Since second surgery is not a valid option for the majority of patients, alternatives to first-line chemotherapy are the ultimate source of hope in OC treatment. In this research work, our aim was to establish a double-resistant HGSC cell line. We used a cell line with intrinsic resistance to Carboplatin and OVCAR8, and induced PTX resistance by pulse and continuous drug exposure (39). Both methods resulted in the generation of two PTX-resistant cell lines presenting over 12-fold resistance when compared to parental OVCAR8 cells (12.3- and 14.5-fold resistance for OVCAR8 PTX R C and OVCAR8 PTX R P, respectively). Interestingly, when looking at long-term survival (using the clonogenic assay), we saw that these values were even higher, with the PTX-resistant cells being over 18-fold more resistant to PTX than their parental cells, which is particularly relevant in the biological tumor context. In addition, in our models, PTX-resistance phenotype was stable in long-term cultures without PTX medium supplementation and following freeze/thaw cycles. This stability of acquired resistance is a guarantee of reliability in this type of *in vitro* tools commonly used to study drug resistance mechanisms and drug discovery (39, 50).

The combination of therapies with independent mechanism of action, such as platinum and taxane drugs, is widely used in oncology in order to minimize the evolution of drug resistance. Some reports suggest that Cisplatin-resistant cells are also cross-resistant to anti-microtubules drugs, such as PTX (51–53). Recently, Patel et al. generated two Cisplatin-resistant cell lines and found that the less Cisplatin-resistant cell line (OVCAR8-CP1) maintained the level of resistance to PTX while the more Cisplatin-resistant cell line (OVCAR8-CP5) presented a significantly higher resistance to anti-microtubule drugs (54). In this report, the authors associate high levels of Cisplatin-resistance with cross-resistance to PTX and demonstrate that this phenomenon is mediated by increased levels of pro-survival TNF/NFkB signaling I (54). However, other authors did not observe cross-resistance between oncology drugs with different mechanisms of action (55). These contradicting findings demonstrate that there is a lot to improve in order to clarify the mechanism behind cross-resistance. As far as we know, the OVCAR8 cell line was obtained from a patient subjected to high-dose Carboplatin. Our results show that OVCAR8 PTX R variants maintained a high resistance level to Carboplatin, not presenting cross-resistance. Similar results were reported by Vaidyanathan et al., which established a PTX-resistant OC cell line model (A2780pacR) without interfering with Carboplatin resistance (56). Analyzing pre-clinical and clinical literature investigating the relationship between platinum and taxane resistance, Stordal et al. observed that there is a positive interaction between taxanes and platinum with residual cross-resistance (14). They also concluded that the inverse relationship between platinum and taxanes resistance seen in cell lines is mirrored in the clinical response to these agents in OC (14), emphasizing that resistant cellular models are valuable in predicting response to chemotherapy and useful to identify new therapeutic targets.

To better characterize the phenotype of these new PTX-resistant variants, we performed a series of functional assays. Our results show that OVCAR8 PTX R variants have similar levels of proliferation and cell viability in culture, maintaining the apoptotic level, anoikis resistance, and migratory capacity when compared to the parental OVCAR8 cell line. OVCAR8 is a Carboplatin-resistant cell line, with an intrinsic high proliferation index, resistance to anoikis and clonogenic survival, and migration capacity (37, 40). These results demonstrate that the main features of the parental OVCAR8 cell line were maintained and the induced PTX resistance did not intensify these original cellular characteristics. These results contrast with some studies, observing an increased migratory capacity induced on chemoresistant variants (57). It has been reported that, at the ultrastructural level, PTX-resistant cells frequently present morphological alterations in mitochondria due to the accumulation of reactive oxygen species (58). The careful inspection of cellular organelles revealed that OVCAR8 PTX R variants and the OVCAR8 cell line display similar mitochondrial morphology. Therefore, the induced PTX resistance did not alter the biological behavior or parental cell line morphology.

Since PTX is an antimitotic agent that binds to β-tubulin and affects microtubule dynamics, PTX-treated cells undergo G2/M arrest, which ultimately leads to apoptosis (59–61). We showed that the mechanism of cell survival in OVCAR8 PTX R variants relied on the acquisition of a new cancer hallmark, an enhanced drug efflux capacity, avoiding drug retention and G2/M arrest. This cell death escape capacity is well reported in several PTX-resistant cell lines (62). The most common mechanism of PTX resistance is the overexpression of drug transporters of the ATP-binding cassette family, promoting cellular drug efflux (49, 63). P-gp is one of these drug efflux pumps being expressed in a variety of normal tissues, such as brain, liver, kidney, placenta, and intestine (64), and having a protective action against xenobiotic substances and toxic compounds (63). In the cancer setting, P-gp is highly expressed in ovary, colon, kidney, adrenocortical, and hepatocellular tumors (65–68) and is correlated with multidrug resistance phenotype (67). In breast and ovarian carcinomas, high P-gp expression levels are associated with the lack of chemotherapy response and poor prognosis (63, 69, 70). Our results show that the expression levels of P-gp in parental OVCAR8 are residual and OVCAR8 PTX R variants present a significant increase in P-gp expression, i.e., more that 75% of cells have a strong membrane staining observed by immunocytochemistry. This switch in P-gp expression profile was observed in other OC cell lines following PTX induction (56) and it is reported that this increase in protein levels is mediated by gene overexpression (14).

Verapamil is a P-gp inhibitor that increases the efficacy of PTX *in vitro* in several cancer cell lines (71, 72). It is well known that P-gp inhibition and/or decreased expression can reverse PTX resistance (19). Our results show a significantly increased accumulation of RH-123 in OVCAR8 PTX R variants in the presence of Verapamil. These results suggest that following P-gp inhibition, cells retain PTX, leading to a decreased cell viability and apoptosis induction. Despite some contradictory data (56), it was previously shown that following P-gp inhibition, PTX-resistant cell lines maintained the high levels of P-gp expression, suggesting that the inhibitory effects were the result of a direct inhibition of the efflux pump and not related to changes in protein expression levels (63). In line with these results, we observed that OVCAR8 PTX R variants maintained the P-gp high expression levels following an inhibition molecule treatment (data not shown).

The inhibition of P-gp is a therapeutic strategy to re-sensitize tumor cells to chemotherapeutic drugs, such as PTX. The importance of this strategy is implicit in the development of a great number of P-gp inhibitors (73) and some of them reached clinical trials (NCT00001302, NCT00001383, and NCT00001944) but with some efficacy limitations. We generated two HGSC cell lines with induced PTX resistance and P-gp overexpression suitable to test new and more effective P-gp inhibitors. Moreover, since OVCAR 8 has intrinsic Carboplatin resistance, our two OVCAR8 PTX R models are also apt to test the efficacy of drugs to revert platinum-taxane resistance. The generation of powerful tools to study chemoresistance in HGSC setting is crucial to discover effective drugs for the treatment of double-resistant tumors. Our goal for future studies is to use these double-chemoresistant cell models to test the capacity of non-oncology drugs to re-sensitize cells to first-line chemotherapy drugs.

## Data Availability Statement

The original contributions presented in the study are included in the article/**Supplementary Material**. Further inquiries can be directed to the corresponding author.

## Author Contributions

MN and SR contributed to conceptualization and design of the study. MN and SR organized the database. MN, PS, CP, and AR performed the experiments and statistical analysis. MN wrote the first draft of the manuscript. MN, PS, RC, CP, AR, HB, GA, and SR wrote sections of the manuscript. All authors contributed to manuscript revision, read, and approved the submitted version.

## Funding

This work was developed at i3S/IPATIMUP, an Associate Laboratory of the Portuguese Ministry of Science, Technology and Higher Education, and partially supported by Fundação para a Ciência e a Tecnologia (FCT). This research was supported by European Regional Development Funds (ERDF) funds through the COMPETE 2020–Operational Program for Competitiveness and Internationalization (POCI), Portugal 2020, Fundação para a Ciência e a Tecnologia (FCT)/Ministério da Ciência, Tecnologia e Inovação (MCTES), under the project POCI 01-0145-FEDER-029503 (PTDC/MEC-ONC/29503/2017) and CESPU (Cooperativa de Ensino Superior Politécnico e Universitário) under the project ComeTarget_CESPU_2017 (to HB). MN acknowledges FCT/MCTES and UE for financial support through a PhD fellowship (2020.04720.BD) co-sponsored by Fundo Social Europeu (FSE) through Programa Operacional Regional Norte (Norte 2020).

## Conflict of Interest

The authors declare that the research was conducted in the absence of any commercial or financial relationships that could be construed as a potential conflict of interest.

## Publisher’s Note

All claims expressed in this article are solely those of the authors and do not necessarily represent those of their affiliated organizations, or those of the publisher, the editors and the reviewers. Any product that may be evaluated in this article, or claim that may be made by its manufacturer, is not guaranteed or endorsed by the publisher.
