# Corrigendum: Generation of Two Paclitaxel-Resistant High-Grade Serous Carcinoma Cell Lines With Increased Expression of P-Glycoprotein

**DOI:** 10.3389/fonc.2022.853608

**Published:** 2022-02-04

**Authors:** Mariana Nunes, Patrícia M. A. Silva, Ricardo Coelho, Carla Pinto, Albina Resende, Hassan Bousbaa, Gabriela M. Almeida, Sara Ricardo

**Affiliations:** ^1^ Differentiation and Cancer Group, Institute for Research and Innovation in Health (i3S) of the University of Porto/Institute of Molecular Pathology and Immunology of the University of Porto (Ipatimup), Porto, Portugal; ^2^ Institute of Biomedical Sciences Abel Salazar (ICBAS), University of Porto, Porto, Portugal; ^3^ CESPU, Institute of Research and Advanced Training in Health Sciences and Technologies (IINFACTS), Gandra, Portugal; ^4^ TOXRUN, Toxicology Research Unit, University Institute of Health Sciences, Polytechnic and University Cooperative (CESPU), Gandra, Portugal; ^5^ Ovarian Cancer Research, Department of Biomedicine, University Hospital Basel and University of Basel, Basel, Switzerland; ^6^ Interdisciplinary Centre of Marine and Environmental Research (CIIMAR), University of Porto, Porto, Portugal; ^7^ Expression Regulation in Cancer Group, Institute for Research and Innovation in Health (i3S) of the University of Porto/Institute of Molecular Pathology and Immunology of the University of Porto (Ipatimup), Porto, Portugal; ^8^ Faculty of Medicine from University of Porto (FMUP), Porto, Portugal

**Keywords:** high-grade serous carcinoma, ovarian cancer, chemoresistance, Paclitaxel, P-glycoprotein

In the original article, we neglected to include the funder. The correct Funding statement appears below. “This work was developed at i3S/IPATIMUP, an Associate Laboratory of the Portuguese Ministry of Science, Technology and Higher Education, and partially supported by Fundação para a Ciência e a Tecnologia (FCT). This research was supported by European Regional Development Funds (ERDF) funds through the COMPETE 2020–Operational Program for Competitiveness and Internationalization (POCI), Portugal 2020, Fundação para a Ciência e a Tecnologia (FCT)/Ministério da Ciência, Tecnologia e Inovação (MCTES), under the project POCI 01-0145-FEDER-029503 (PTDC/MEC-ONC/29503/2017) and CESPU (Cooperativa de Ensino Superior Politécnico e Universitário) under the project ComeTarget_CESPU_2017 (to HB). MN acknowledges FCT/MCTES and UE for financial support through a PhD fellowship (2020.04720.BD) co-sponsored by Fundo Social Europeu (FSE) through Programa Operacional Regional Norte (Norte 2020).”

In the published article, there was an error in affiliation 4. Instead of “TOXRUN, Toxicology Research Unit, University Institute of Health Sciences, Advanced Polytechnic and University Cooperative (CESPU), Gandra, Portugal” it should be “TOXRUN, Toxicology Research Unit, University Institute of Health Sciences, Polytechnic and University Cooperative (CESPU), Gandra, Portugal”.

The authors apologize for these errors and state that this does not change the scientific conclusions of the article in any way. The original article has been updated.

## Publisher’s Note

All claims expressed in this article are solely those of the authors and do not necessarily represent those of their affiliated organizations, or those of the publisher, the editors and the reviewers. Any product that may be evaluated in this article, or claim that may be made by its manufacturer, is not guaranteed or endorsed by the publisher.

