# Supplementary material for: Generation of Two Paclitaxel-Resistant High-Grade Serous Carcinoma Cell Lines With Increased Expression of P-Glycoprotein
Source: Front Oncol. 2021 Oct 21;11:752127. doi: 10.3389/fonc.2021.752127 (PMC8566917; doi:10.3389/fonc.2021.752127)
Supplement: Supplementary file 1 [file Image_1.pdf]

Supplementary Material

A

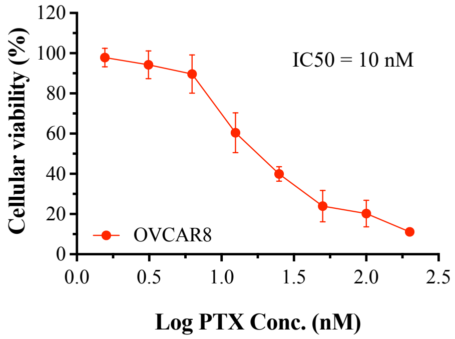

B

Fold resistance	Initial	Persistent culture	Freeze/thawing cycles
**OVCAR8 PTX R C**	12.271	14.878	14.587
**OVCAR8 PTX R P**	14.537	14.676	20.810

Supplementary Figure 1. IC50 and Fold-Resistance for PTX in OVCAR8 and OVCAR8 PTX R cell lines. (**A**) Line chart showing cell viability and IC50 for PTX in OVCAR8 cells obtained by PB assay after exposure to PTX (1.56 to 200 nM) for 48 hours. (**B**) Table showing PTX fold-resistance for OVCAR8 PTX R cell lines. All assays were done in triplicate in at least three independent experiments. Data is expressed as mean ± standard deviation and plotted using GraphPad Prism Software Inc. v6.

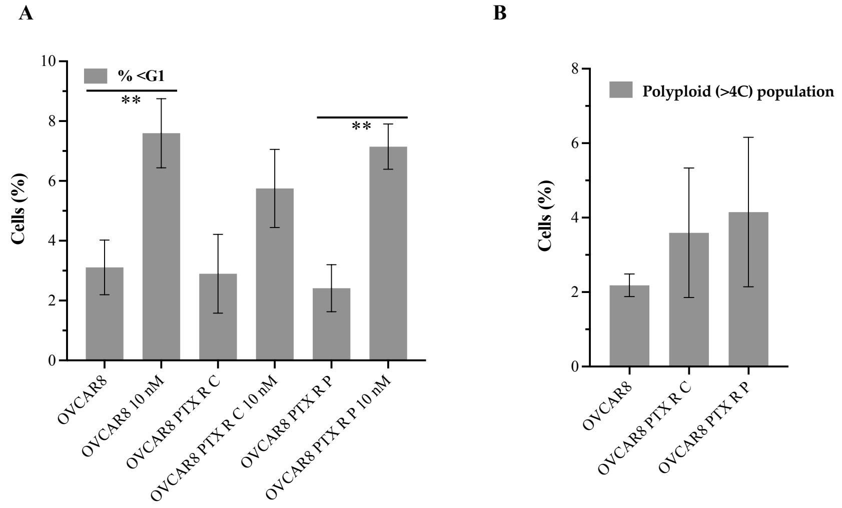

Supplementary Figure 2. Proportion of Sub-G1 and polyploid (>4C) populations in OVCAR8 and OVCAR8 PTX R cell lines. (**A**) Bar chart for cell debris and apoptotic cells (%<G1) of OVCAR8 and OVCAR8 PTX R cell lines using propidium iodide (PI) staining, after exposure to 10 nM PTX during 24 hours. (**B**) Bar chart showing percentage of polyploid (>4C) populations of OVCAR8 and OVCAR8 PTX R cell lines using propidium iodide (PI) staining. Flow cytometry was performed using BD FACS Canto™ II (BD Biosciences) flow cytometer and data was analyzed by FlowJo software v10.0.7. Assay was done in triplicate in at least three independent experiments. Data is expressed as mean ± standard deviation and plotted using GraphPad Prism Software Inc. v6. Statistical analysis was performed using ordinary one-way ANOVA followed by two-way ANOVA followed by Turkey's multiple comparison test (**A** and **B**) and values of *<0.05; **<0.001; ***<0.0005; ****<0.0001 as considered statistically significant.
